# Achieving high mobility, low-voltage operating organic field-effect transistor nonvolatile memory by an ultraviolet-ozone treating ferroelectric terpolymer

**DOI:** 10.1038/srep36291

**Published:** 2016-11-08

**Authors:** Lanyi Xiang, Wei Wang, Wenfa Xie

**Affiliations:** 1 State Key Laboratory on Integrated Optoelectronics, College of Electronic Science and Engineering, Jilin University, 2699 Qianjin Street, Changchun 130012, China

## Abstract

Poly(vinylidene fluoride–trifluoroethylene) has been widely used as a dielectric of the ferroelectric organic field-effect transistor (FE-OFET) nonvolatile memory (NVM). Some critical issues, including low mobility and high operation voltage, existed in these FE-OFET NVMs, should be resolved before considering to their commercial application. In this paper, we demonstrated low-voltage operating FE-OFET NVMs based on a ferroelectric terpolymer poly(vinylidene-fluoride-trifluoroethylene-chlorotrifluoroethylene) [P(VDF-TrFE-CTFE)] owed to its low coercive field. By applying an ultraviolet-ozone (UVO) treatment to modify the surface of P(VDF-TrFE-CTFE) films, the growth model of the pentacene film was changed, which improved the pentacene grain size and the interface morphology of the pentacene/P(VDF-TrFE-CTFE). Thus, the mobility of the FE-OFET was significantly improved. As a result, a high performance FE-OFET NVM, with a high mobility of 0.8 cm^2 ^V^−1 ^s^−1^, large memory window of 15.4~19.2, good memory on/off ratio of 10^3^, the reliable memory endurance over 100 cycles and stable memory retention ability, was achieved at a low operation voltage of ±15 V.

Organic memories have attracted great research interest for their many advantages over their inorganic counterparts, such as low cost, mechanical flexibility, solution processability and low-temperature fabrication^1^,^2^,^3^. Among many configurations of organic memories, a nonvolatile memory (NVM) based on ferroelectric organic field-effect transistor (FE-OFET) has attracted more attention due to its non-destructive read out, short programming time and nonvolatile data storage^1^,^4^. The memory mechanism is due to the modulation of the channel conductance by the remanent polarization of ferroelectric dielectrics after the supplied programming (P) and erasing (E) voltage. In previous reports, the ferroelectric copolymer poly(vinylidene fluoride–trifluoroethylene) [P(VDF-TrFE)] was the most widely used dielectric in FE-OFET NVMs. In the last decade, the performances of FE-OFET NVMs have been improved. Nevertheless, the utilization of P(VDF-TrFE) to achieve a FE-OFET NVM still suffers from some major challenges. For instance, the rough surface of the crystalline P(VDF-TrFE) film usually leads to low field-effect mobility (*μ*) of 10^–4^~10^−2 ^cm^2 ^V^−1 ^s^−1^ in FE-OFET NVMs^1^,^5^,^6^,^7^,^8^,^9^. The low mobility induces a relatively slow charge accumulation in response to the gate voltage (*V*_*G*_), which limits the operation frequency of the devices^8^,^10^,^11^. On the other hand, the high coercive field of 50 MV/m in P(VDF-TrFE) usually brings high P/E voltage (>40 V) in previous reported FE-OTFT NVMs^1^,^5^,^9^,^12^,^13^,^14^,^15^. An additional buffer layer, such as poly(vinyl cinnamate) or poly(methyl methacrylate), has been employed to improve the mobility in FE-OFET^11^,^16^,^17^. However, it made the problem of high P/E voltage severer because the inserted buffer layer with a low dielectric constant shared the supplied P/E voltage^11^,^16^. And, another negative effect is that the inserted buffer layer often diminishes the remanant polarization of a ferroelectric dielectric in the FE-OFET and thus the non-volatile memory capability becomes disappeared^9^. Although using thinner P(VDF-TrFE) film could decrease the P/E voltage, extra problems, such as reduction in polarization, increasing in switching filed, switching time and leakage current occurs, as the ferroelectric polymer film thickness reduces^18^,^19^,^20^,^21^. Additionally, the spin-coated P(VDF-TrFE) films were processed with dimethylformamide (DMF), 2-butanone (MEK), or cyclohexanone as a solvent in all previous reports, most likely due to its high solubility. However, DMF is carcinogenic, and the latter two are toxic.

Terpolymer poly(vinylidene-fluoride- trifluoroethylene-chlorotrifluoroethylene) [P(VDF-TrFE-CTFE)], a new ferroelectric polymer developing from the P(VDF-TrFE), has a high dielectric constant (*κ*) of about 28.2, remnant polarization (*P*_*r*_) of 12.9 mC/m^2^ at a electric field of 150 MV/m and a low coercive field of 14.3 V/μm (These data is got from the material specification by Piezotech-Arkema Corp), which is critical to realize a low-voltage operating FE-OFET NVM. And, it is an important way to reduce the operation voltage in OFETs by employing a gate dielectric with a high *κ*^22^,^23^. Furthermore, P(VDF-TrFE-CTFE) can well dissolve in butyl acetate, which is greener and safer than DMF, MEK or cyclohexanone, considering the negative effects of the solvents on the environment and the safety of staffs. Up to now, the FE-OFET NVM based on ferroelectric P(VDF-TrFE-CTFE) as the gate dielectric has rarely reported^21^.

In this work, we demonstrated a high mobility, low-voltage operating FE-OFET NVM based on an ultraviolet-ozone (UVO) treating P(VDF-TrFE-CTFE) dielectric, which was spin-coated from a solution in butyl acetate. Our work is promising to overcome the above problems. After the P(VDF-TrFE-CTFE) film was treated under an UVO atmosphere for 60 min, the following depositing pentacene grew at a two-dimensional layer-by-layer mode. As a result, large pentacene grain size and good interface morphology at pentacene/P(VDF-TrFE-CTFE) were achieved, which had a critical influence on improving the mobility. At last, good memory characteristics, with high mobility of 0.8 cm^2 ^V^−1^ s^−1^, low P/E voltage of ±15 V, large memory window (*ΔV*_*T*_) of about 15.4~19.2 V, good memory on/off ratio of 10^3^, the reliable endurance over 100 cycles and stable retention ability, were achieved in our FE-OFET.

## Experimental

**Figure 1:**
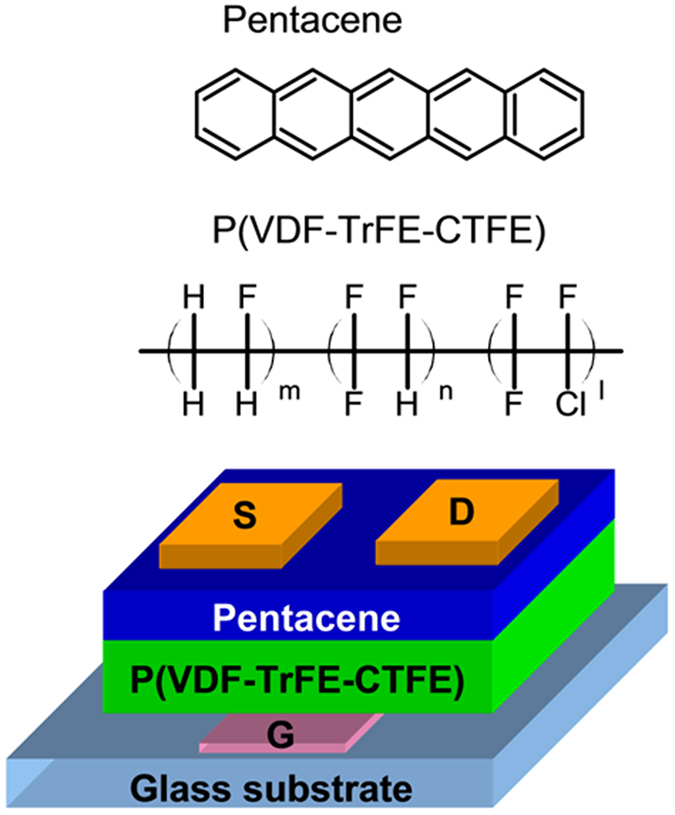
Schematic diagram of present FE-OFETs and the chemical structures of the ferroelectric and semiconductor materials used in this work.

All FE-OFET devices were fabricated on the patterned indium tin oxide (ITO) coated glass substrates, which act as a gate electrode. After the substrates were routinely cleaned and dried, the P(VDF-TrFE-CTFE) with a composition of 64.2/27.1/8.7 mol% (purchased from Piezotech-Arkema Corp., France) was spin coated on the ITO gate electrode as a gate dielectric, from a solution of 7 wt % in butyl acetate. Then, the P(VDF-TrFE-CTFE) films were annealed at 120 °C for 120 min, which is lower than the typical annealing temperature near 140 °C for the formation of the ferroelectric β-phase of P(VDF-TrFE)^1^,^5^,^6^,^7^,^8^,^9^,^10^,^14^,^15^. The resulted film thicknesses of P(VDF-TrFE-CTFE) is about 580 nm, measured by a Dektak 6 surface profiler. Prior to the deposition of the semiconducting pentacene layers, the UVO treatment was carried out on the P(VDF-TrFE-CTFE) films at a power of 28 mW/cm^2^ for 0~60 min, respectively. A 40-nm-thick pentacene film was thermally deposited on the P(VDF-TrFE-CTFE) films at a speed of 0.2 Å/s. Finally, top-contact source-drain (S-D) electrodes of MoO_3_ (8 nm) and Cu (60 nm) were thermally deposited and patterned by a shadow mask. The channel length (*L*) and width (*W*) were 140 and 2000 μm, respectively. The schematic diagram of our FE-OFET devices and the chemical structures of the ferroelectric and semiconductor materials used in this work are shown in Fig. 1. Additional, the capacitor with a structure of ITO/P(VDF-TrFE-CTFE) (580 nm)/Cu was also prepared. The electrical properties of the FE-OFETs were measured by a semiconductor parameter analyzer (Agilent B2902) in the ambient atmosphere at room temperature. The ferroelectric hysteresis properties and the capacitance of the P(VDF-TrFE-CTFE) film were measured by P-PMF1213–346 ferroelectric test system (Radiant Technology, U.S.A.) and ZL-5 model LCR analyzer, respectively. The surface morphologies of both the P(VDF-TrFE-CTFE) films and the pentacene films were characterized by tapping-mode atomic force microscopy (AFM) (Dimension Icon, Bruker Co.). The x-ray diffraction (XRD) was performed by using a Bruker D8 Advance X, Pert diffractmeter (Cu-Ka: λ = 1.540 Å).

## Results and Discussion

**Figure 2:**
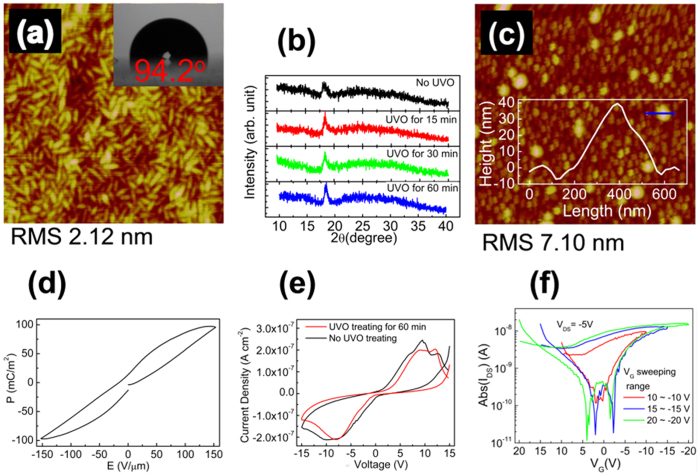
(**a**) The AFM image of the P(VDF-TrFE-CTFE) film without UVO treatment (2 μm × 2 μm). (**b**) XRD patterns of the P(VDF-TrFE-CTFE) films with or without UVO treatment. (**c**) The AFM image of the pentacene film deposited on the P(VDF-TrFE-CTFE) film without UVO treatment (5 μm × 5 μm). (**d**) Polarization-electric field hysteresis properties for ITO/P(VDF-TrFE-CTFE)/Au ferroelectric capacitors. (**e**) The *I*–*V* characteristics of the P(VDF-TrFE-CTFE) films with or without UVO treatment. (**f**) The linear transfer characteristics of the FE-OFET based on the P(VDF-TrFE-CTFE) film without UVO treatment. The inset of (**a**) shows the water contact angle on the P(VDF-TrFE-CTFE) film without UVO treatment. The inset of (**c**) shows the morphological profile of the blue lined region in the image.

The P(VDF-TrFE-CTFE) film without UVO treatment exhibited the characteristic randomly distributed rod-like microdomains, which were about 150 nm and 40 nm in length and width, respectively, as shown in Fig. 2a. The root-mean-square (RMS) roughness of the P(VDF-TrFE-CTFE) film was about 2.1 nm. The XRD pattern of the P(VDF-TrFE-CTFE) film without UVO treatment shows a prominent peak at a 2θ angle of 18°, indicating the (002, 020) reflection faces of ferroelectric phase (Fig. 2b)^24^. The pentacene film deposited on the P(VDF-TrFE-CTFE) film without UVO treatment exhibited an inferior morphology, with a RMS roughness of 7.10 nm and many small dot-like grains with an average diameter of about 100 nm, as shown in Fig. 2c. Moreover, some towery island-like aggregation were also observed with a height of 20~40 nm, diameter of 200~400 nm and density of about 1.6 per square micrometer, as shown by the inset of Fig. 2c.

Figure 2d shows the ferroelectric hysteresis properties of the P(VDF-TrFE-CTFE) film with the remanent polarization and coercive filed were about 13.6 mC/m^2^ and 14.8 V/μm, respectively, which are approximate to the data supplied by Piezotech-Arkema Corp. The *I*–*V* characteristics of the capacitor exhibited a low leakage current density of 10^−7 ^A cm^−2^ at a voltage sweeping range of ±15 V (Fig. 2e), indicating a good electric insulator property in our P(VDF-TrFE-CTFE) film, which is required for the stable operation of a FE-OFET. The transfer characteristics of a typical FE-OFET based on a P(VDF-TrFE-CTFE) film without UVO treatment were measured at the drain-source voltage (*V*_*DS*_) of −5 V, with different *V*_*G*_ sweeping ranges from ±10 V to ±20 V, as shown in Fig. 2f. The present FE-OFET operated in p-channel accumulation mode and exhibited a counter-clockwise hysteresis behaviour. The hysteresis loops increased with the extending of the *V*_*G*_ sweeping range, and almost saturated at the *V*_*G*_ sweeping range of ±15 V. The hysteresis characteristics were attributed to the ferroelectric properties of P(VDF-TrFE-CTFE), which agreed well with the operation mechanism reported in many FE-OFETs based on a ferroelectric P(VDF-TrFE) gate dielectric^1^,^5^,^6^,^7^,^8^,^9^,^10^,^11^,^12^,^13^,^14^,^15^,^16^,^17^. The hysteresis characteristics mean a promising application of present device as a memory. However, low on state drain-source current (*I*_*DS*_) of ~10^−8^ A was observed in present FE-OFET, which led to a low memory on/off ratio of about 20 at a reading voltage (*V*_*R*_) of *V*_*G*_ = 0 V and the *V*_*G*_ sweeping rang of ±15 V. The linear mobility (*μ*_*lin*_) was extracted using the conventional metal-oxide-semiconductor field-effect transistor mode described in equation 1, by the transconductance, *g*_*m*_, in the linear region according to equation 2,


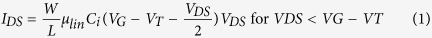



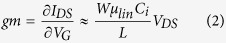


where, the *C*_*i*_ is the gate dielectric layer capacitance per unit area, and *V*_*T*_ is the threshold voltage^25^. The *C*_*i*_ of about 40.5 nF cm^−2^ was calculated from the measured data of the capacitor at the constant voltage of 1 V and frequency of 1000 Hz. The *V*_*T*_ can be extracted by extrapolating the transfer curves in the linear region, with the intercept on the x-axis (*I*_*DS*_ was zero) indicating the values of (*V*_*T*_ + *V*_*DS*_/2). The low linear mobility of 4.2~6.8 × 10^−4 ^cm^2 ^V^−1 ^s^−1^ of present FE-OFET was responsible for its low on state *I*_*DS*_. The memory on/off ratio is one of the important parameters of the memory, defining as the ratio of *I*_*DS*_ at both reading 1 and 0 states. The poor pentacene morphology was one of the reasons for the bad mobility in present FE-OFET device. High mobility is desired because it can directly enlarge the on current and thus the memory on/off ratio, which is favorable for distinguishing the Boolean 0 and 1 state, especially for that after a long storage time.

**Figure 3:**
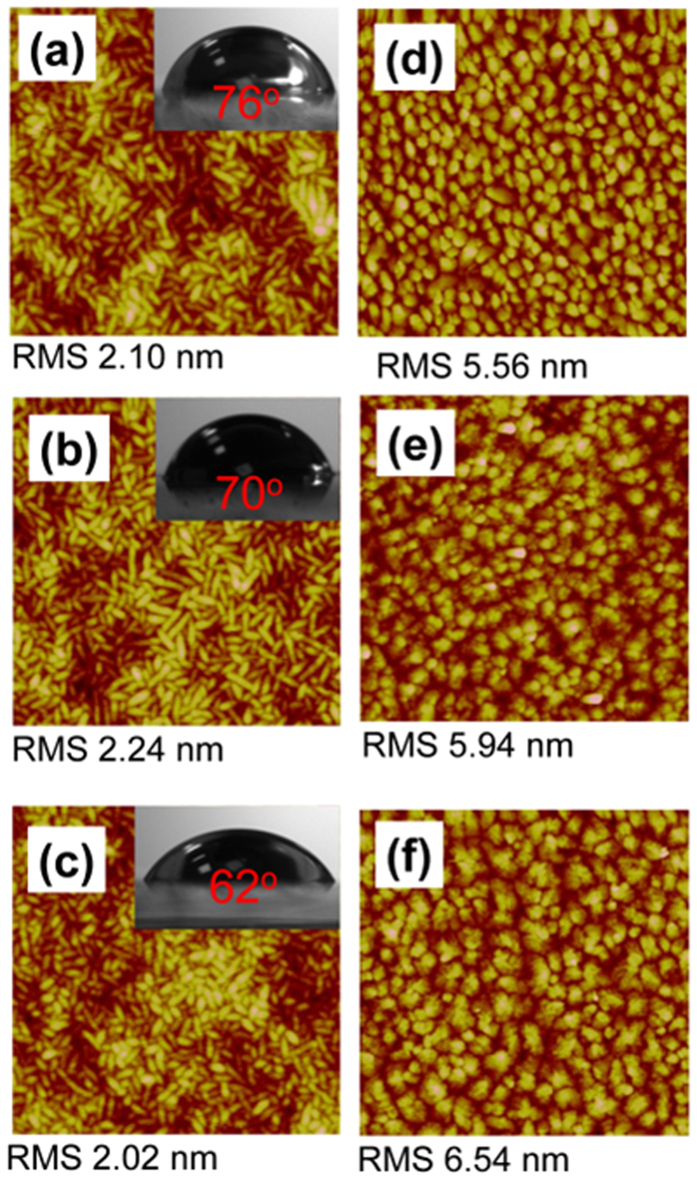
The AFM images of the P(VDF-TrFE-CTFE) films (2 μm × 2 μm) with the UVO treatment time of (**a**) 15, (**b**) 30 and (**c**) 60 min, respectively. The AFM images of pentacene films (5 μm × 5 μm) deposited on the P(VDF-TrFE-CTFE) films with a UVO treatment time of (**d**) 15, (**e**) 30 and (**f**) 60 min, respectively. The insets of (a)-(c) show the corresponding water contact angle of each P(VDF-TrFE-CTFE) film.

Motivated by previous reports that utilizing UVO treatment on the dielectrics improved the deposited pentacene morphology, which had an obviously influence on the mobility of OFET^26^,^27^. Here, we researched the influence of the UVO treating P(VDF-TrFE-CTFE) on the mobility of the FE-OFET. As shown in Fig. 3a–c, the AFM images demonstrate that UVO treated the P(VDF-TrFE-CTFE) films showed similar rod-like microdomains and surface morphology with similar RMS values. On the other hand, the XRD patterns of the UVO treated P(VDF-TrFE-CTFE) films also showed similar characteristics (Fig. 2b). Both AFM and XRD characterizations indicated that the UVO treatment did not change the surface morphology and microstructure of the P(VDF-TrFE-CTFE) films.

The morphology of the deposited pentacene films showed an obviously dependence on the time of the UVO treatment on the under P(VDF-TrFE-CTFE) films, as shown in Fig. 3d–f. For the pentacene film on the P(VDF-TrFE-CTFE) film treated by UVO for 15 min, it exhibited a uniform polycrystalline microstructure with an average grain size of about 180 × 200 nm^2^ and a surface RMS roughness of about 6.0 nm (Fig. 3d). As the time of the UVO treatment on the P(VDF-TrFE-CTFE) films increased to 30 and 60 min, the crystalline quality of the deposited pentacene films was further improved, exhibiting a uniform dendritic polycrystalline microstructure and larger grains with an average size of about 250 × 350 and 400 × 450 nm^2^, respectively (Fig. 3e,f).

**Figure 4:**
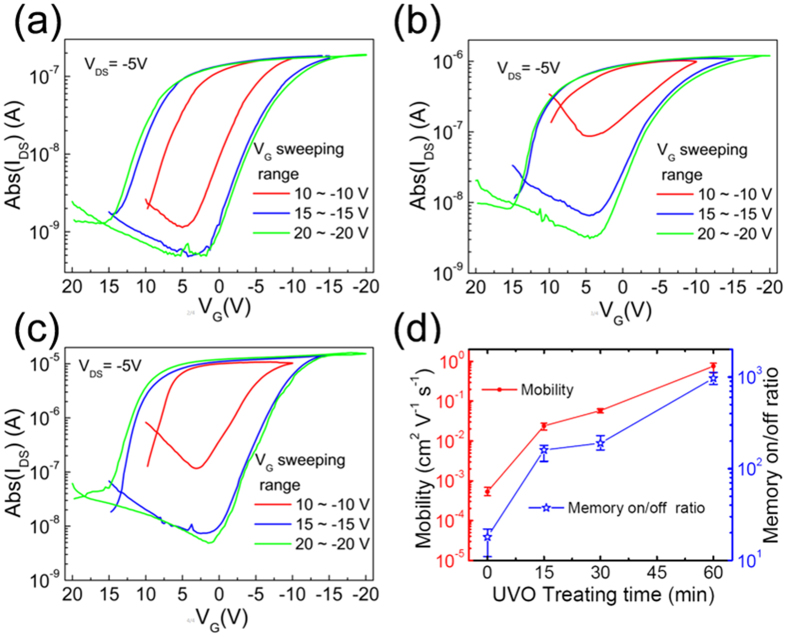
The linear transfer characteristics of the FE-OFETs based on the P(VDF-TrFE-CTFE) dielectric modified by UVO treatment for (**a**) 15, (**b**) 30 and (**c**) 60 min, respectively, measured at different *V*_*G*_ sweeping ranges. (**d**) The evolution of the mobility and the memory on/off ratio of the FE-OFETs with the time of UVO treatment on the P(VDF-TrFE-CTFE).

The transfer characteristics of the FE-OFETs with a UVO treating P(VDF-TrFE-CTFE) dielectric were also measured at the *V*_*DS*_ of −5 V and different *V*_*G*_ sweeping ranges from ±10 V to ±20 V, as shown in Fig. 4a–c. It is clear that the on state *I*_*DS*_ of the FE-OFETs obviously increased with the increasing time of the UVO treatment on the P(VDF-TrFE-CTFE) films. However, the *V*_*T*_ of devices was not affected by the UVO treatment on the P(VDF-TrFE-CTFE) films. Both the linear mobility and the memory on/off ratio, extracted from ten devices for every sample, were summarized in Fig. 4d, as the function of the UVO treating time. The both obviously increased with the time of the UVO treatment on the P(VDF-TrFE-CTFE) films. The increasing mobility resulted in the increase of the on state *I*_*DS*_, which directly promoted the increase of the memory on/off ratio. As a result, high mobility with an average value of 0.8 cm^2 ^V^−1 ^s^−1^ was achieved in the FE-OFET with a 60 min UVO treating P(VDF-TrFE-CTFE), which were three orders of magnitude higher than that of the FE-OFET without UVO treatment. In present high mobility FE-OFET, memory on/off ratio of 10^3^ was obtained, which was similar to that in some recent reports^6^,^7^,^8^,^11^,^14^. The better pentacene morphology with larger crystalline grains was one of the reasons to achieve higher mobility in our FE-OFETs for the decreasing grain boundaries, which acts as trap sites in carrier transport and limit the carrier mobility in channel. The similar results have been reported in other OFETs based on a polycrystalline small-molecule organic semiconductor^28^,^29^.

In general, charge carriers transport in the first few molecular monolayer of the channel adjacent to the dielectric layer^30^,^31^. Thus, the interface properties of pentacene/P(VDF-TrFE-CTFE) were investigated to further understand the dependence of the mobility in the FE-OFETs on the time of the UVO treatment on the P(VDF-TrFE-CTFE) films. The water contact angel on the P(VDF-TrFE-CTFE) films with different UVO treating time were measured, as shown in the insets of Figs 2a and 3a–c and Table 1. The decreasing water contact angel indicated that the surface energy of the P(VDF-TrFE-CTFE) films increased with the time of the UVO treatment (Table 1). And, the increased polar component of the surface energy made the surface of P(VDF-TrFE-CTFE) films more polar. The evolution of the surface energy of the P(VDF-TrFE-CTFE) had an obvious influence on the pentacene growth, which resulted in different interface properties of pentacene/P(VDF-TrFE-CTFE).

**Figure 5:**
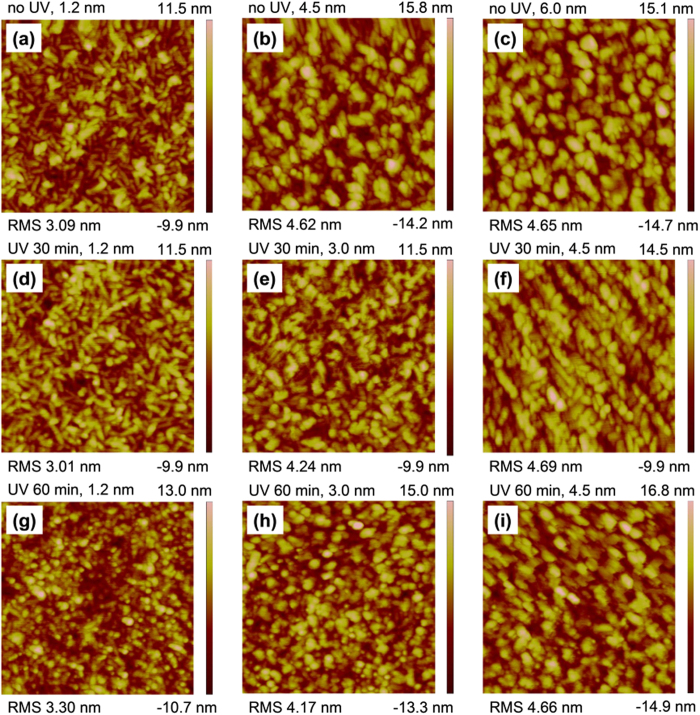
AFM images of pentacene films formation at various thickness on the P(VDF-TrFE-CTFE) films with or without UVO treatment. All images area are 2 μm × 2 μm in size.

On the surface of P(VDF-TrFE-CTFE) films with a surface energy much lower than that of pentacene (42.1 mJ cm^−2^)^26^, the deposited pentacene molecules try to minimize the total surface energy of system by reducing the interfacial area. So, these molecules aggregated on the surface area, rather than covered the whole surface area. As for the pentacene film with an average thickness of 1.2 nm (~0.8 monolayer) deposited on the surface of P(VDF-TrFE-CTFE) film without UVO treatment, a low coverage was observed with many bared rod-like P(VDF-TrFE-CTFE) crystalline microdomains, as shown in Fig. 5a. The subsequently deposited pentacene molecular followed a three-dimensional island-like growth mode. Even for the pentacene film with an average thickness of 4.5 nm (~3.0 monolayers), some rod-like P(VDF-TrFE-CTFE) crystalline microdomains were still bared (Fig. 5b). It approximated to get a full coverage of pentacene film on the P(VDF-TrFE-CTFE) film when its thickness increased to 6.0 nm (Fig. 5c).

With the increase of the surface energy on the P(VDF-TrFE-CTFE), the pentacene growth mode exhibited a transition from a three-dimensional island-like mode to a two-dimensional layer-by-layer mode (Fig. 5d–f). The coverage area of the initial deposited 1.2 nm thick pentacene film on the 30 min UVO treating P(VDF-TrFE-CTFE) was obviously larger than that on the P(VDF-TrFE-CTFE) film without UVO treatment (Fig. 5d). An approximate full coverage of pentacene on the 30 min UVO treating P(VDF-TrFE-CTFE) film was observed when its thickness increased to 4.5 nm (Fig. 5f).

On the surface of the P(VDF-TrFE-CTFE) film with a 60 min UVO treatment, pentacene molecules were ready adhesion on the dielectric rather than aggregation each other, due to the similar surface energy (41.1 mJ cm^−2^) with that of the pentacene film. As a result, many dot-like islands firstly formed and then extended at a two-dimensional planar direction with the increasing thickness in the initial stage of pentacene growth, as shown in Fig. 5g–i. No more than two monolayers, a full coverage of pentacene on the P(VDF-TrFE-CTFE) film was obtained (Fig. 5h). This two-dimensional layer-by-layer mode of pentacene growth on the 60 min UVO treating P(VDF-TrFE-CTFE) film resulted in a continuous pentacene monolayer adjacent to the dielectric and large dendritic crystalline grains. As a result, a continuous charge carrier transporting channel and a greatly reduced trap density at the pentacene/P(VDF-TrFE-CTFE) interface were achieved, which resulted in a greatly enlarged mobility.

The UVO treatment has been extensively applied to modify the surface chemistry and wetting characteristics of many natural or synthetic polymer surfaces^27^,^32^,^33^,^34^,^35^. The exposure of organic polymer to UV radiation can lead to a dissociative excitation of C–C or C–H bonds in the near-surface layer^35^. Upon oxidation of polymer surfaces by atomic oxygen and ozone originating from the UV radiation through the abstraction of hydrogen atoms from the polymer chains, producing radical carbon sits, hydroxyl (−OH) is generally introduced on the polymer surface^27^,^32^,^33^,^34^, which has been identified as electron traps^36^. It was also possible that −OH groups were created on the surface of the P(VDF-TrFE-CTFE) by UVO treatment, which resulted in the increase of the polar component of the surface energy and enhancement of the surface hydrophilicity (Table 1 and Fig. 3). On the other hand, many electrons were trapped by −OH, which induced many holes accumulated in the channel at the off state and resulted in a visible off current during the first measurement of *V*_*G*_ sweeping from +10 V to −10 V (Fig. 4a–c). At the supplied negative *V*_*G*_, these electrons were detrapped from −OH groups to the channel and were neutralized with the accumulating holes. Thus, the sufficient low off current was obtained at the next measurement of bidirectional *V*_*G*_ sweeping between ±15 and ±20 V, respectively, due to no additional holes accumulating in the channel at the off state (Fig. 4a–c).

The *I*–*V* characteristics of the capacitor of ITO/P(VDF-TrFE-CTFE) (580 nm, UVO treatment for 60 min)/Cu were similar to that of the capacitor without UVO treatment (Fig. 2e), indicating that the UVO treatment did not weaken the electric insulator property of P(VDF-TrFE-CTFE) films. It is noted that the large saturated memory window (*ΔV*_*T*_) of 15.4~19.2 V was achieved at the *V*_*G*_ sweeping range of ±15 V in all FE-OFETs, which was not dependent on the time of the UVO treatment on the P(VDF-TrFE-CTFE) films. The *ΔV*_*T*_ is another important parameter in the memory, and is defined as the difference between the *V*_*T*_ in the forward and backward sweeps of the *V*_*G*_. The results demonstrated that our FE-OFET memories can well operate at low P/E voltage of ±15 V, which is similar to the P/E voltage used in previous reported low-voltage operating FE-OFET memories based on a thin P(VDF-TrFE) layer^37^,^38^,^39^. And, the ratio of the *ΔV*_*T*_ versus the P/E voltage in our FE-OFET memories was larger than 50%, which was larger than that of 25~40% obtained in the most previous reported FE-OFET memories^1^,^4^,^5^,^6^,^7^,^8^,^9^,^10^,^11^,^12^,^13^,^14^,^15^,^16^,^17^,^21^,^31^,^37^,^38^.

**Figure 6:**
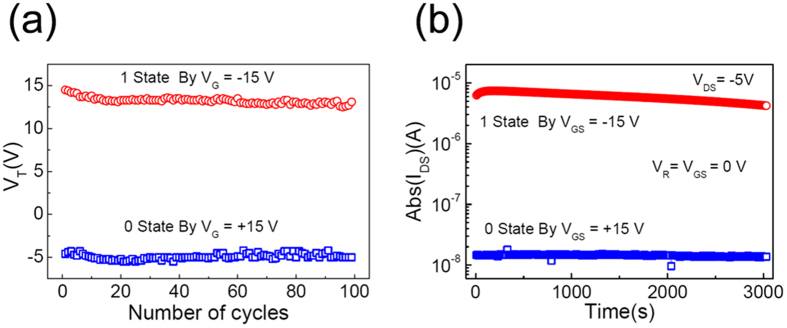
The switching endurance and the time-dependent retention characteristics of the FE-OFET memory with a P(VDF-TrFE-CTFE) dielectric by UVO treatment for 60 min.

Figure 6 shows The Boolean system 0/1 state switching endurance and the data storage retention ability of the high mobility FE-OFET memory with a 60 min UVO treating P(VDF-TrFE-CTFE) film. The positive *V*_*T*_ of about 14.5 V and the negative *V*_*T*_ of about −4.6 V were achieved after supplied the P/E voltage of *V*_*G*_ = ± 15 V for 0.1 s, which corresponded to the Boolean system 0/1 state, respectively. During the periods of P/E operation, the *V*_*DS*_ was kept at 0 V. The present memory exhibited a good stability with the slightly fluctuation of *V*_*T*_ at both 0 and 1 states over 100 P/E switching cycles (Fig. 6a), which was comparable to that of some recently reported FE-OFET memories with a P(VDF-TrFE) dielectric^8^,^14^,^15^,^21^. The reliability of present device was evaluated by time-dependent data retention ability. After supplied P/E voltage of *V*_*G*_ = ± 15 V for 0.1 s, the corresponding reading *I*_*DS*_ at both 0 and 1 states was recorded as the function of time at a time interval of 10 s at *V*_*R*_ = *V*_*G*_ = 0 V and *V*_*DS*_ = −5 V, respectively. The reading *I*_*DS*_ at both 0 and 1 states maintained well during the measurement range of 3000 s, and the memory on/off ratio exhibited a slightly delay, which declined to 72% of its initial value at the end of measurement (Fig. 6b), which was comparable to that of some recently reported FE-OFET memories with a P(VDF-TrFE) dielectric^7^,^8^,^17^. The results indicated that our high mobility FE-OFET had good memory parameters, with low P/E voltage, large memory window, good memory on/off ratio, stable and reliable operating ability.

## Conclusions

In summary, we demonstrated a high mobility, low-voltage operating FE-OFET memory based a ferroelectric terpolymer P(VDF-TrFE-CTFE), which processes a much lower coercive voltage compared with the widely used copolymer P(VDF-TrFE). The surface energy on the P(VDF-TrFE-CTFE) film increased with the time of UVO treatment, which caused the change of the pentacene growth model. The large pentacene grain and good interface morphology of pentacene/P(VDF-TrFE-CTFE) were observed at the case of 60 min UVO treatment on the P(VDF-TrFE-CTFE) dielectric. As a result, a high performance FE-OFET memory was achieved, with high mobility of 0.8 cm^2 ^V^−1 ^s^−1^, low P/E voltage of ±15 V, large memory window of 15.4~19.2 V, good memory on/off ratio of 10^3^, the reliable switching endurance over 100 cycles and stable memory retention ability.

## Additional Information

**How to cite this article**: Xiang, L. *et al*. Achieving high mobility, low-voltage operating organic field-effect transistor nonvolatile memory by an ultraviolet-ozone treating ferroelectric terpolymer. *Sci. Rep.* **6**, 36291; doi: 10.1038/srep36291 (2016).

**Publisher’s note:** Springer Nature remains neutral with regard to jurisdictional claims in published maps and institutional affiliations.

## Figures and Tables

**Table 1:** Surface energy of P(VDF-TrFE-CTFE) films.

UVO treating time (min)	Contact angle (^o^)		*γ*_*s*_^*p*^mJ m^−2^ (a)	*γ*_*s*_^*d*^mJ m^−2^ (a)	*γ*_*s*_mJ m^−2^ (b)
	Water	Toluene			
0	94.2	4.0	1.7	27.4	29.1
15	76.0	3.6	10.1	23.3	33.4
30	70.0	3.2	14.2	22.1	36.3
60	62.0	2.6	20.5	20.6	41.1

(a)*γ*_*s*_^*p*^ and *γ*_*s*_^*d*^ are the polar and dispersion components of the surface energy, respetively. (b) *γ*_*s*_ is the surface energy of P(VDF-TrFE-CTFE) films. *γ*_*s*_ = *γ*_*s*_^*p*^ + *γ*_*s*_^*d*^.
